# Firefly (Coleoptera, Lampyridae) species from the Atlantic Forest hotspot, Brazil

**DOI:** 10.3897/BDJ.11.e101000

**Published:** 2023-03-23

**Authors:** Stephanie Vaz, Mariana Mendes, Gabriel Khattar, Margarete Macedo, Cristina Ronquillo, Alejandra Zarzo-Arias, Joaquín Hortal, Luiz Silveira

**Affiliations:** 1 Universidade Federal do Rio de Janeiro, Rio de Janeiro, Brazil Universidade Federal do Rio de Janeiro Rio de Janeiro Brazil; 2 Concordia University, Montreal, Canada Concordia University Montreal Canada; 3 Museo Nacional de Ciencias Naturales (MNCN-CSIC), Madrid, Spain Museo Nacional de Ciencias Naturales (MNCN-CSIC) Madrid Spain; 4 Universidad de Oviedo, Asturias, Spain Universidad de Oviedo Asturias Spain; 5 Dept. Biogeography & Global Change, BEI-MNCN-CSIC, Madrid, Spain Dept. Biogeography & Global Change, BEI-MNCN-CSIC Madrid Spain; 6 Universidade Federal de Goiás, Goiânia, Brazil Universidade Federal de Goiás Goiânia Brazil; 7 Faculdade de Ciências da Universidade de Lisboa, Lisboa, Portugal Faculdade de Ciências da Universidade de Lisboa Lisboa Portugal; 8 Western Carolina University, Cullowhee, United States of America Western Carolina University Cullowhee United States of America

**Keywords:** Neotropics, dataset, species occurrences, endemism, rainforest, South America

## Abstract

**Background:**

We compiled a database of firefly species records from the Atlantic Forest hotspot in Brazil and made it available at GBIF. Data were gathered from literature and from several key entomological collections, including: Coleção entomológica Prof. José Alfredo Pinheiro Dutra (DZRJ/UFRJ) and Coleção do Laboratório de Ecologia de Insetos from Universidade Federal do Rio de Janeiro (CLEI/UFRJ); Coleção Entomológica do Instituto Oswaldo Cruz (CEIOC); Museu de Zoologia da Universidade de São Paulo (MZSP); Coleção Entomológica Pe. Jesus Santiago Moure from Universidade Federal do Paraná (DZUP/UFPR); and Coleção Entomológica from Universidade Federal Rural de Pernambuco (UFRPE). This database represents the largest contribution to a public repository of recorded occurrences from Neotropical fireflies.

**New information:**

This dataset shows the occurrence and abundance of firefly species in the Atlantic Forest hotspot. Firefly species endemic to this biome are also present and considered in the study. These data can assist scientific and societal needs, by supporting future research projects and conservation decision-making.

## Introduction

The Atlantic Forest covers seventeen of twenty-six Brazilian States and occupies almost the whole country's coast (Oliveira and Engel 2017). It is recognised as one of the thirty-six hotspots in the world and is a global priority for biodiversity conservation (Esser et al. 2019). Unfortunately, the Atlantic Forest also represents the biome with the highest number of fauna and flora species threatened by urban sprawl and land-use change. Landscape modification and climate change are strong drivers of habitat loss and changes in correlated ecosystem services (Maldaner et al. 2021). Due to this, the Atlantic Forest is one of the biomes most threatened by climate change (Scarano and Ceotto 2015). The evident shifts in temperature and precipitation patterns (Pereira et al. 2010) have started to induce responses on several species of plants and animals (Root et al. 2003, Parmesan 2006, VanDerWal et al. 2012). Thus, the Atlantic Forest is one of the habitats highly prone to the adverse effects of climate change, jeopardising its biodiversity and endemic species richness. According to the Intergovernmental Panel on Climate Change, the vulnerability to climate change disruption in a system is extreme (I.P.C.C. 2014). In this scenario, it is important to anticipate the effects of climate change in the ecosystems, in order to decrease its damage by building up possible solutions, aligned with mitigation. These strategies can reduce the vulnerability of species and increase the resilience of natural and human systems, aiming to avoid or minimise these negative impacts (I.P.C.C. 2014).

Fireflies (Lampyridae) are amongst the many organisms threatened by anthropogenic stressors and climate change in the Atlantic Forest (Vaz et al. 2021, Khattar et al. 2022). They are a cosmopolitan family of about 2,200 species worldwide (Branham 2010), with most of its known diversity found in the Neotropics and the Asian Southeast (Lawrence and Newton 1995). This number of species is largely underestimated due to severe taxonomic impediments, in addition to a lack of targeted sampling and experts, especially in the Neotropics (McDermott 1966, Silveira and Mermudes 2013). Indeed, Neotropical fireflies need massive nomenclatural and curatorial work, which are of utmost importance to facilitate their research and conservation.

Besides their relevance, fireflies are very charismatic for society (Fallon et al. 2021, Prischmann-Voldseth 2022). They are present in music, poems, paintings and other kinds of arts, being used as a tool to foster conservation of nature through environmental education (Lenko and Papavero 1997, Faust 2004). Furthermore, they play a significant role in the economy of many countries as flagship species in ecotourism (Lewis et al. 2020). In addition, they are sensitive to light pollution and other forms of environmental degradation (Viviani et al. 2010, Owens et al. 2022), being important bioindicators of ecosystems’ health (Fallon et al. 2021). Lastly, their medical importance is relevant since they prey on intermediate hosts of waterborne diseases (Viviani 1989).

Even though anthropogenic threats have been investigated, the extent to which climate and land use changes impact fireflies has never been quantitatively assessed anywhere in South America. An important way to protect fireflies from these threats is to protect forest remnants at the Atlantic Forest hotspot, since protected areas act as a shelter for thousands of species. In this regard, mobilising the biodiversity data currently stored in natural history collections and literature can be particularly useful to conduct large-scale analyses in macroecology and conservation, provided that its coverage is adequately assessed and field inventories are conducted to fill in the gaps that are eventually identified (Hortal et al. 2007, Ronquillo et al. 2020).

Shortfalls, particularly in species identities and distribution (Linnean and Wallacean shortfalls, respectively, see Hortal et al. (2015)), hampers the elaboration of predictive models that could provide information on the needs for management of firefly populations. In this regard, one of the main concerns about fireflies is the absence of occurrence records publicly available in repositories; therefore making it difficult to obtain any assessment on their status. Thus, this work aims to compile and to make a public dataset of firefly diversity in Brazil, which would be of high relevance to advance our knowledge of this particular taxa, especially when applying predictive models which could be the base for new and expert-knowledge-based conservation and management actions. To do this, we evaluated and gathered data from collections and fieldwork, as well as from a revision of literature. In addition, this dataset will support the project described below, which aims to propose conservation policies for the Atlantic Forest, identifying threats and predicting effects of global change on firefly communities, sustaining and providing a better global perspective on potential firefly extinctions in South America.

## Project description

### Title

Keep the forest shiny: mapping threats to provide information for conservation planning of firefly species endemic to the Atlantic Forest hotspot

### Personnel

Stephanie Vaz, Mariana Mendes, Gabriel Khattar, Margarete Macedo, Cristina Ronquillo, Alejandra Zarzo-Arias, Joaquín Hortal, Luiz Felipe Silveira

### Funding

Coordenação de Aperfeiçoamento de Pessoal de Nível Superior 88887.694716/2022-00

## Sampling methods

### Study extent

**Figure 1.**
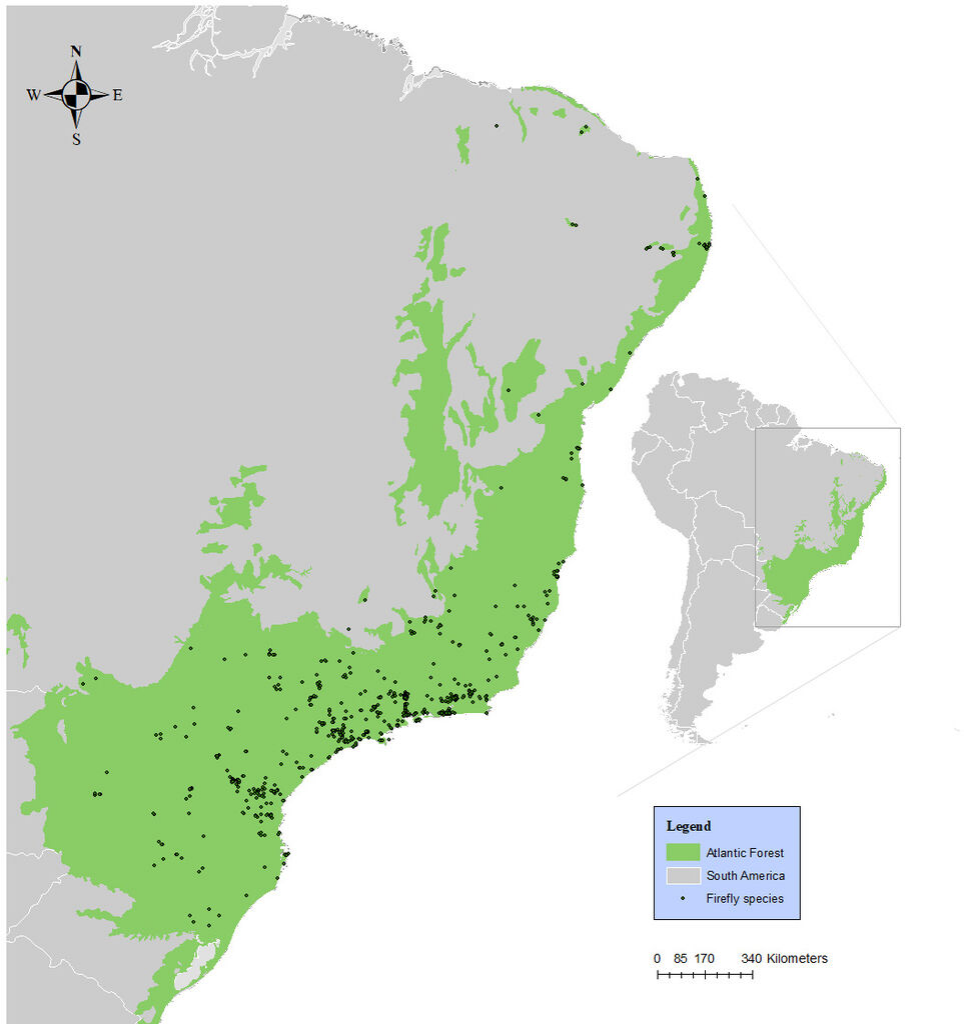
Fireflies distribution in the Atlantic Forest hotspot.

Information on lampyrid specimens included in this study was obtained from scientific collections, literature and unpublished data from fieldwork research over nine years throughout the Brazilian Atlantic Forest. The samples were evaluated from different areas in the Brazilian coast, mainly from the south-eastern and south regions (Fig. 1), which are the most densely inhabited. Our field expeditions were undertaken between 2013 and 2022 and encompassed the Parque Nacional da Serra dos Órgãos, Parque Nacional da Tijuca, Parque Estadual da Ilha Grande and Parque Estadual da Pedra Branca, all placed within Rio de Janeiro State. The scientific collections digitised contained records of fireflies sampled from the 1885s to the present.

### Sampling description

**Scientific collections**

- Universidade Federal do Rio de Janeiro - Coleção entomológica Prof. José Alfredo Pinheiro Dutra from Universidade Federal do Rio de Janeiro (DZRJ), Rio de Janeiro, Brazil.
- Universidade Federal do Rio de Janeiro - Coleção do Laboratório de Ecologia de Insetos from Universidade Federal do Rio de Janeiro (CLEI), Rio de Janeiro, Brazil.
- Fiocruz/CEIOC - Coleção Entomológica do Instituto Oswaldo Cruz, Rio de Janeiro, Brazil.
- Universidade Federal do Parana - Coleção Entomológica Pe. Jesus Santiago Moure from Universidade Federal do Paraná (DZUP/UFPR), Paraná, Brazil.
- Museu de Zoologia da Universidade de Sao Paulo (MZUSP), São Paulo, Brazil.
- Universidade Federal Rural de Pernambuco - Coleção entomológica da Universidade Federal Rural de Pernambuco (UFRPE), Pernambuco, Brazil.

Firefly species were analysed under a stereomicroscope and we imaged characters relevant to species identification. All of them were compared to the species-type material and original descriptions, when available. For specimens whose identity could not be confirmed at the species level, we included a morphotype code (“sp1”, “sp2” and so on). This code was standardised by the first author, so all specimens with the same code in the database pertain to the same morphotype. Entomological institutions and their collections are discriminated as provided by GBIF. Museu Nacional/UFRJ institution code holds both Coleção entomológica Prof. José Alfredo Pinheiro Dutra from Universidade Federal do Rio de Janeiro (DZRJ) and Coleção do Laboratório de Ecologia de Insetos from Universidade Federal do Rio de Janeiro (CLEI) collection codes in this paper.

**Fieldwork**

**Figure 2.**
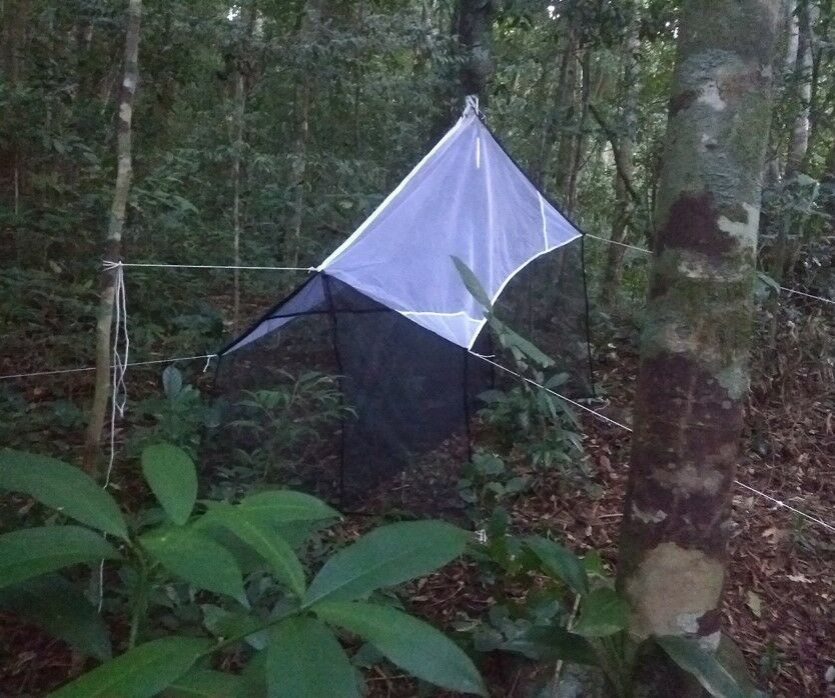
Typical Malaise trap in the Atlantic Forest.

The surveys at Parque Nacional da Serra dos Órgãos, Parque Nacional da Tijuca, Parque Estadual da Pedra Branca and Parque Estadual da Ilha Grande were conducted following the same protocol. We used Malaise traps (Fig. 2) and active search across all habitat types and seasons, to enhance the coverage and completeness of our sampling effort. This fieldwork methodology (Suppl. material 1) increased the overall success of the surveys (see Silveira et al. (2020)).

**Literature**

Most of the data incorporated into this dataset came from our team, which includes two leading lampyrid taxonomists: Stephanie Vaz and Luiz Silveira. Together, SV and LS have been studying and collecting fireflies since 2010 and helped to build one of the most comprehensive and well-curated firefly collections in the Americas. This work includes: (i) the original descriptions of many of the endemic species covered in this dataset and (ii) monographic reviews which confirmed the endemism (to the Atlantic Forest) of several other species. Data were taken from the original source (Silveira and Mermudes 2013, Silveira and Mermudes 2014a, Silveira and Mermudes 2014b, Bocakova et al. 2016, Silveira et al. 2016, Silveira and Mermudes 2017, Silveira et al. 2018, Campello-Gonçalves et al. 2019, Nunes et al. 2019, Silveira et al. 2019, Silveira et al. 2020, Vaz et al. 2020a, Vaz et al. 2020b, Silveira et al. 2021, Vaz et al. 2021a, Bocakova et al. 2022 and Campello-Gonçalves et al. 2022).

### Quality control

All records were validated geographically and taxonomically. Coordinates were plotted and revised to verify the geographical location and locality. All scientific names were checked for types and matched to the species information.

### Step description

**Figure 3.**
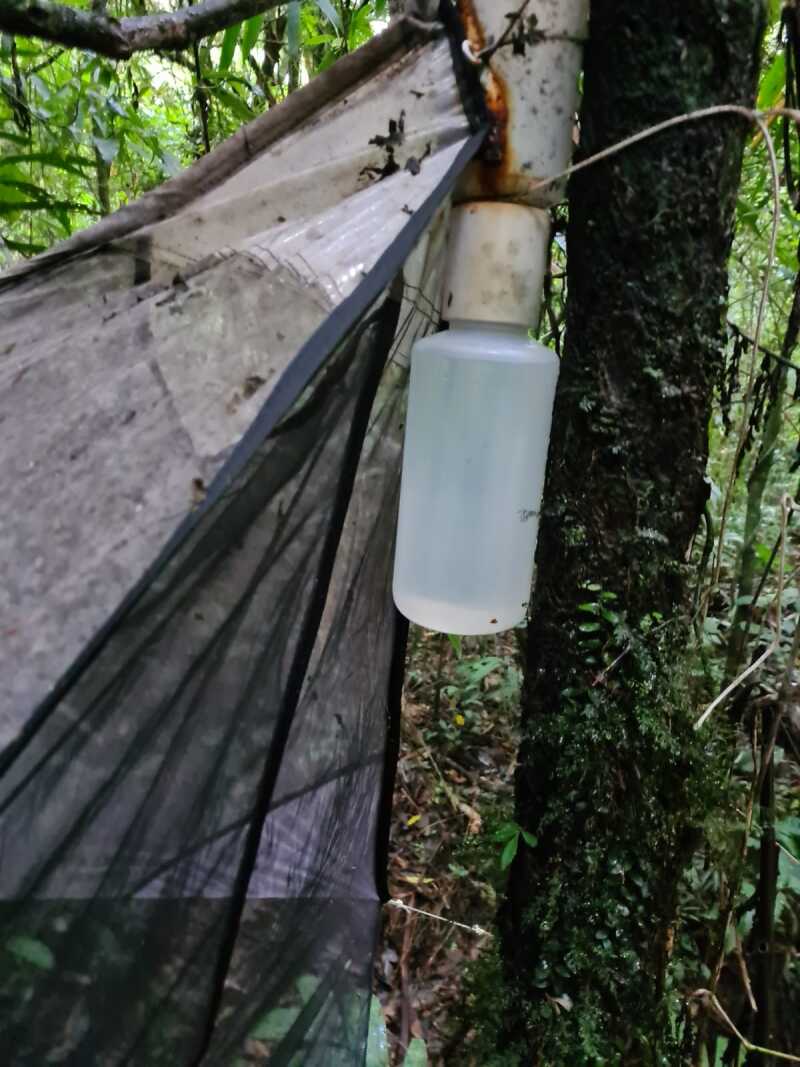
Ethanol > 90% recipient to preserved specimens from our fieldwork.

Most specimens deposited in collections were preserved in entomological drawers under dry conditions. The information contained on the specimens’ labels was collected to build the dataset. Specimens from our fieldwork are preserved in ethanol > 90% since the date of collection (Fig. 3), which better preserves soft-bodied insects, such as fireflies and also prevents DNA degradation.

## Geographic coverage

### Description

**Figure 4.**
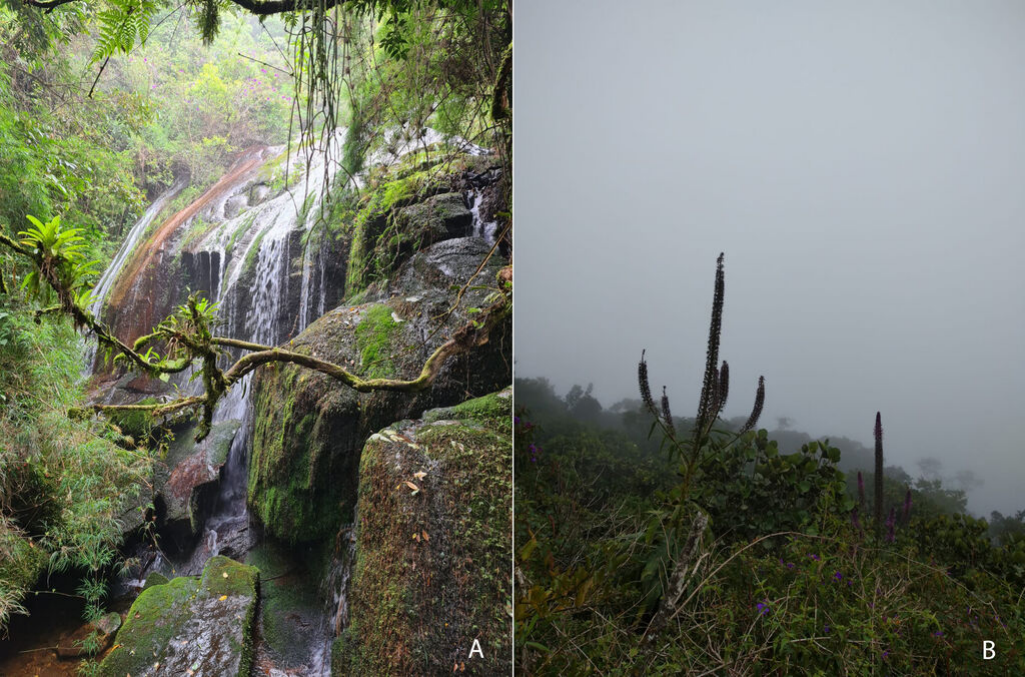
The Brazilian Atlantic Forest - (A) Cachoeira do Papel - Trilha da Pedra do Sino, Teresópolis, Rio de Janeiro - Brazil (B) Mirante - Trilha da Pedra do Sino, Teresópolis, Rio de Janeiro - Brazil.

The samples were collected from different areas throughout the Brazilian Atlantic Forest (Fig. 4). The data obtained from entomological collections was georeferenced by using the information in specimens’ labels stored in appropriate drawers or jars.

### Coordinates

-29.78 and -4.11 Latitude; and -54.95 and -34.88 Longitude.

## Taxonomic coverage

### Description

The dataset consists in 3010 records, 36 genera and 107 different species. Additionally, there are 362 records with genus taxonomic status comprising 44 morphotypes which could not be identified at species level with 100% certainty. That means 153 potential species in this dataset representing 7.65% of the species recorded worldwide (see Branham (2010)), although it is necessary to bear in mind that the total number of Lampyridae species worldwide is still underestimated (Silveira and Mermudes 2013).

The most frequently reported species was *Photurisfemoralis* Curtis, 1839, which was recovered from 377 locations. *Photinusfrater* (Olivier, 1905) was the second most abundant, found at 334 locations, followed by *Lucidotaflabellicornis* Fabricius, 1781 (122), *Amydetesapicalis* Germar, 1824 (104) and *Photinusluna* Curtis, 1932 (101).

The five most abundant species were from the subfamily Photurinae and Lampyrinae. The full firefly dataset was recorded from 14 ecoregions in the Atlantic Forest.

## Temporal coverage

### Notes

1885–2019

## Usage licence

### Usage licence

Other

### IP rights notes

The dataset in the current work is licensed under a Creative Commons Attribution (CC-BY) 4.0 License.

## Data resources

### Data package title

Firefly (Coleoptera: Lampyridae) species from the Atlantic Forest hotspot, Brazil

### Resource link

https://www.gbif.org/dataset/f2fafb08-f21e-4d6c-82f7-ed3463ffa03c

### Alternative identifiers

https://ipt.sibbr.gov.br/mnrj/resource?r=af_fireflies_br_01

### Number of data sets

1

### Data set 1.

#### Data set name

Firefly (Coleoptera: Lampyridae) species from the Atlantic Forest hotspot, Brazil

#### Data format

Darwin Core Archive format

#### Download URL

https://www.gbif.org/dataset/f2fafb08-f21e-4d6c-82f7-ed3463ffa03c

#### Data format version

Darwin Core Archive format

#### Description

The dataset contains information on fireflies (Coleoptera, Lampyridae) from the Brazilian Atlantic Forest, obtained from our own fieldwork, specimens deposited in scientific collections and data available in literature. The aims and objectives of this project are to build and compile a dataset tailored to: (i) evaluate the coverage and eventual biases of the distributional data available for Atlantic Forest fireflies; and (ii) develop models to provide further insights on how light pollution, land-use and climate changes may affect the firefly species endemic to the Atlantic Forest. The specimens have been lumped into a single dataset, forming the basis of S. Vaz 's PhD thesis. This dataset will be used to investigate priority areas for conservation in the Atlantic Forest and assess the connection amongst firefly populations and communities located in different forest fragments. This dataset is published in GBIF under the licence CC BY 4.0 and the metadata can be also consulted at Vaz et al. (2022). If you have any questions regarding this dataset, please do not hesitate to contact us via the contact information provided in the metadata.

**Data set 1.** 

Column label	Column description
datasetName	The name identifying the dataset from which the record was derived.
occurrenceID	An identifier for the Occurrence (as opposed to a particular digital record of the occurrence). In the absence of a persistent global unique identifier, construct one from a combination of identifiers in the record that will most closely make the occurrenceID globally unique.
basisOfRecord	The specific nature of the data record.
eventDate	The date-time or interval during which an Event occurred. For occurrences, this is the date-time when the event was recorded. Not suitable for a time in a geological context.
year	The four-digit year in which the Event occurred, according to the Common Era Calendar.
month	The integer month in which the Event occurred.
day	The integer day of the month on which the Event occurred.
scientificName	The full scientific name, with authorship and date information, if known. When forming part of an Identification, this should be the name in lowest level taxonomic rank that can be determined.
higherClassification	A list (concatenated and separated) of taxa names terminating at the rank immediately superior to the taxon referenced in the taxon record.
kingdom	The full scientific name of the kingdom in which the taxon is classified.
phylum	The full scientific name of the phylum or division in which the taxon is classified.
class	The full scientific name of the class in which the taxon is classified.
order	The full scientific name of the order in which the taxon is classified.
family	The full scientific name of the family in which the taxon is classified.
genus	The full scientific name of the genus in which the taxon is classified.
specificEpithet	The name of the first or species epithet of the scientificName.
taxonRank	The taxonomic rank of the most specific name in the scientificName.
identificationRemarks	Comments or notes about the Identification.
identifiedBy	A list (concatenated and separated) of names of people, groups or organisations who assigned the Taxon to the subject.
decimalLatitude	The geographic latitude (in decimal degrees, using the spatial reference system given in geodeticDatum) of the geographic centre of a Location. Positive values are north of the Equator, negative values are south of it. Legal values lie between -90 and 90, inclusive.
decimalLongitude	The geographic longitude (in decimal degrees, using the spatial reference system given in geodeticDatum) of the geographic centre of a Location. Positive values are east of the Greenwich Meridian, negative values are west of it. Legal values lie between -180 and 180, inclusive.
geodeticDatum	The ellipsoid, geodetic datum or spatial reference system (SRS), upon which the geographic coordinates given in decimalLatitude and decimalLongitude are based.
georeferencedBy	A list (concatenated and separated) of names of people, groups or organisations who determined the georeference (spatial representation) for the Location.
georeferenceProtocol	A description or reference to the methods used to determine the spatial footprint, coordinates and uncertainties.
continent	The name of the continent in which the Location occurs.
country	The name of the country or major administrative unit in which the Location occurs.
countryCode	The standard code for the country in which the Location occurs.
stateProvince	The name of the next smaller administrative region than country (state, province, canton, department, region etc.) in which the Location occurs.
county	The full, unabbreviated name of the next smaller administrative region than stateProvince (county, shire, department etc.) in which the Location occurs.
locality	Less specific geographic information can be provided in other geographic terms (higherGeography, continent, country, stateProvince, county, municipality, waterBody, island, islandGroup). This term may contain information modified from the original to correct perceived errors or standardise the description.
associatedReferences	A list (concatenated and separated) of identifiers (publication, bibliographic reference, global unique identifier, URI) of literature associated with the Occurrence.
institutionCode	The name (or acronym) in use by the institution having custody of the object(s) or information referred to in the record.
collectionCode	The name, acronym, coden or initialism identifying the collection or dataset from which the record was derived.
recordedBy	A list (concatenated and separated) of names of people, groups or organisations responsible for recording the original Occurrence. The primary collector or observer, especially one who applies a personal identifier (recordNumber), should be listed first.

## Supplementary Material

012E2FD4-8984-5575-A830-FBDE72388377 10.3897/BDJ.11.e101000.suppl1 Supplementary material 1 Malaise trap in Atlantic Forest Data type multimedia Brief description How to collect fireflies by using a Malaise trap. File: oo_776748.mp4 https://binary.pensoft.net/file/776748 Stephanie Vaz et al.
